# A Rare Case of Rosai-Dorfman Disease in an Adult Male Associated with Auto-Immune Hemolytic Anemia

**DOI:** 10.4084/MJHID.2013.022

**Published:** 2013-04-10

**Authors:** Mickey Sachdeva, Haifaa Abdulhaq

**Affiliations:** 1 Internal Medicine, University of California, San Francisco-Fresno, Fresno, California; 2 Hematology/Oncology, University of California, San Francisco-Fresno, Fresno, California

## Abstract

Rosai-Dorfman disease (RDD) is a rare benign histiocytic proliferative disorder predominantly of the lymph nodes, which mostly occurs in children and young adults typically presenting with lymphadenopathy. Our case is of a 63 year-old African-American male who presented with subjective fever, weight loss, bilateral axillary and inguinal lymphadenopathy as well as auto-immune hemolytic anemia. The histological analysis showed emperipolesis and histiocytes that were positive for S-100 and CD-68 consistent with RDD. After steroid treatment and splenectomy, patient’s symptoms and hemolytic anemia had resolved. Our case is the first case of RDD reported to be associated with auto-immune hemolytic anemia in an adult.

## Case Report

Sinus histiocytosis with massive lymphadenopathy (SHML) is a rare histiocytic syndrome first described in 1969 by Rosai and Dorfman hence named after them. It is predominantly a disease of childhood and early adulthood (mean age: 20.6 years). Typically, patients present with cervical and often widespread lymphadenopathy associated with fever and weight loss.^1^,^4^

**Figure 1:**
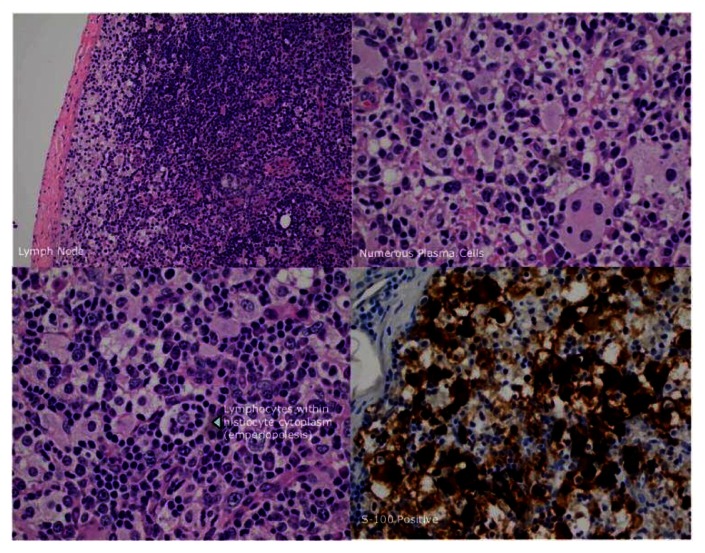


Here we describe a patient with Rosai-Dorfman disease (RDD) who presented in his 60’s with auto-immune hemolytic anemia. A 63 year old African-American male with history of coronary artery and cerebrovascular disease presented to our hospital with generalized weakness, subjective fever and weight loss of 30 pounds in the last 6 months. On physical examination he was found to have bilateral axillary lymphadenopathy, otherwise his exam was unremarkable. Laboratory data were notable for hemoglobin-hematocrit of 4.7 g/dl-18.1%, WBC of 13,000/uL, Platelet count of 432,000/uL, LDH elevated at 600 U/L (normal range 100–230 IU/L), Haptoglobin low at 7 mg/dL, Reticulocyte Index elevated at 13.9%, with positive Direct Coombs test for IgG Antibody, Ferritin of 600 ng/mL (normal range 22–322 ng/mL), Fibrinogen of 564 mg/dL (normal range 190–490 mg/dL) and Triglycerides of 71 mg/dL (normal range 30–158 mg/dL) (Table 1). A CT (computed tomography) scan of the chest, abdomen and pelvis showed massive lymphadenopathy-bilateral axillary, right hilar, mediastinal, retroperitoneal, iliac and inguinal region without splenomegaly. The patient received red packed blood cell transfusions to maintain Hemoglobin above 7 g/dl and was started on methylprednisolone 2 mg/kg intravenously (IV) daily. Differential diagnoses included infectious etiologies such as HIV (human immunodeficiency virus), EBV (Epstein-Barr virus), CMV (Cytomegalovirus); autoimmune disease such as SLE (systemic lupus erythematosus); medications; malignancy such as lymphoma and rare lymphoproliferative disorders such as Rosai-Dorfman disease, Castleman’s disease and hemophagocytic lymphohistiocytosis (HLH. In our patient HIV screening test, EBV and CMV serology was negative. ANA (anti-nuclear antibodies) was slightly positive with titer of 1:40 however Anti-dsDNA was negative and the patient did not clinically meet criteria for diagnosis of SLE. Medications were reviewed and no culprit was revealed. The patient had elevated fibrinogen with elevated ferritin and normal triglyceride level which are not consistent with diagnosis of HLH. Decision was made to pursue an excisional biopsy of an enlarged inguinal lymph node. The histologic analysis of the lymph node showed a proliferation of epitheliod histiocytes. The histiocytes showed abundant pale cytoplasm, containing lymphocytes and plasma cells within their cytoplasm (also known as emperipolesis), histiocytes were positive for S-100 protein and CD68; a hallmark of Rosai-Dorfman disease (Figure 1). The lymphocytes were otherwise unremarkable, CD20 and CD30 showed a normal distribution of B-cells and T-cells; there was no evidence of lymphoma.

The patient’s hemolytic anemia did not improve so he received immunoglobulin at 1 gm/kg IV daily for 2 days. He had a transient response for a few days only. He later received 1 dose of Rituximab at 375 mg/m^2^, however his hemolysis worsened after 2 days of receiving Rituximab and hemoglobin dropped again to 5 g/dl, so he underwent laparoscopic splenectomy. Pathology of the spleen was consistent with infiltration of Rosai-Dorfman disease. Within a few days of splenectomy his hemolytic anemia resolved with normalization of LDH and reticulocyte count, hemoglobin improved to 11.2 g/dl. The patient was discharged home on tapering dose of prednisone for 2 weeks. On follow up the patient’s B symptoms resolved. CT scan of the chest, abdomen and pelvis obtained after 2 months showed decrease in the size of lymphadenopathy by greater than 50%.

Rosai-Dorfman disease is an uncommon idiopathic disease of the young which presents with massive painless lymphadenopathy defined by its histopathological features as mentioned above.

Some cases also present with extranodal manifestations. Involvement of extranodal sites by RDD has been noted since the earliest descriptions of the disease. In a RDD case registry of 423 cases, 43% of patients were found to have at least one site of extranodal involvement. Skin and soft tissue is the most common site of extranodal RDD,^2^,^6^ followed by the nasal cavity/paranasal sinuses. There has been extranodal involvement reported nearly in every tract of the human body, including the skeletal system.^3^ There is also evidence of some cases presenting with autoimmune phenomena which was associated with an unfavorable prognosis. In the same registry of RDD cases, the most common autoimmune phenomena were hematologic autoantibodies (usually directed against red cell antigens) and joint disease. The joint disease varied from mild polyarthralgia to disabling arthritis.^3^

The etiology and pathogenesis of the disease is unclear. The increased incidence of serum auto-immune antibodies during active disease suggests a possible correlation with an immune dysregulatory process.^2^ Although associations with various herpes virus infections have been reported, these most likely represent detection of lymphocytes or macrophages harboring these viruses with no relation to etiology. A possible model for the key histopathologic finding, emperipolesis of lymphocytes by macrophages has been reported. It has been hypothesized that macrophage-activating cytokines stimulate the macrophages to ingest lymphocytes.^1^ This phenomenon of emperipolesis can also be seen in diseases such as autoimmune hepatitis, lymphoma, hemophagocytic lymphohistiocytosis and malignant histiocytosis.^5^ The presence of benign histiocytes with emperipolesis, absence of cellular atypia, immunohistochemical profile, and associated clinical and laboratory features distinguish Rosai-Dorfman disease from these other disorders.

Many cases are self-limited and do not require therapy. Surgery may be useful for symptomatic treatment of large lymph nodes. Most patients will have a slow but steady decrease in the size of their lymph nodes over months to years. Multiorgan involvement or dysfunction, and association with immune dysfunction are poor prognostic indicators and indicate the necessity of treatment. Several therapies have been used including glucocorticoids and chemotherapy with success in some cases. Because no clinical trials have been done, there is no standard chemotherapy regimen. Treatment has been based on anecdotal reports and the most common chemotherapeutic agents are Methotrexate & 6-mercaptopurine.^1^

While 18 of the 423 cases in the previously mentioned registry were pediatric cases of auto-immune hemolytic anemia associated with RDD, our case, to our knowledge is the first case in the literature of auto-immune hemolytic anemia associated with RDD to be reported in the adult population.^3^

## Figures and Tables

**Table 1:** Laboratory Data

Variable	Reference Range	Hospital Admission	Hospital Discharge
Hematocrit (%)	40 – 54	18.1	35.3
Hemoglobin (g/dL)	14.0 – 18.0	4.7	11.2
White-cell count (per mm^3^)	4,000 – 11,000	13,000	16,200
Platelet count (per mm^3^)	140,000 – 440,000	432,000	287,000
Mean corpuscular volume (fL)	80 – 99	111	98
Reticulocytes (%)	0.5 – 2.0	28	14.6
Ferritin (ng/mL)	22–322	600	
Fibrinogen (mg/dL)	190–490	564	
LDH (IU/L)	100–230	600	282
Total Bilirubin (mg/dL)	0.3 – 1.2	2.3	1.9
Unconjugated bilirubin (mg/dL)	0.0 – 1	1.6	1.2
Haptoglobin (mg/dL)	40 – 240	7	
Direct Coombs		+ IgG	
HIV (human immunodeficiency virus)		Negative	
ANA (Anti-Nuclear Antibodies)		Positive	
-ANA titer	< 1:40	1:40	
-ANA (Immuno- fluorescence pattern)		Homogenous	
-dsDNA Antibody IgG		0	
-Smith Antibody IgG		0	
Hepatitis C Antibody		Negative	
Hepatitis B Surface Antigen		Negative	
Hepatitis B Core Antibody		Negative	
Triglycerides (mg/dL)	30–158	71	
